# Long-term mind-body exercise enhances cognitive control over working memory-driven attentional capture

**DOI:** 10.3389/fpsyg.2026.1775012

**Published:** 2026-02-26

**Authors:** Biye Cai, Guoping Liu, Wu Jiang, Zonghao Zhang

**Affiliations:** 1 School of Physical Education and Sports Science, Soochow University, Suzhou, China; 2 Jiangsu Urban and Rural Construction Vocational College, Changzhou, China

**Keywords:** attentional guidance, cognitive control, distractor suppression, mind–body exercise, working memory

## Abstract

Internal representations in working memory (WM) automatically bias attention toward matching stimuli, a process termed WM-driven attentional capture that is modulated by cognitive control. Although previous studies have found that mind–body exercise has a promoting effect on cognitive control, the effect of mind–body exercise on resistance to proactive interference from working memory has yet to be investigated. The present study used the classic WM/visual search dual-task paradigm to investigate the effects of baduanjin mind–body exercise on WM-driven attentional capture. Sixty-seven healthy college students were randomly allocated to a training group or a control group. The training group underwent 16 weeks of baduanjin intervention at a frequency of 3 days/week for 60 min/session. The results showed that mind–body exercise significantly reduced reaction times (RTs) in both visual search and WM tasks. More importantly, the training group showed a significant reduction in WM-driven attentional capture during the posttest phase compared with the same measure during the pretest phase. However, the control group showed no such improvement. These results indicated that mind–body exercise could modulate WM-driven attentional capture, potentially attributable to strengthened top-down cognitive control and optimized allocation of attentional resources. The present study provides preliminary evidence that long-term mind–body exercise improves the young individuals’ ability to resist proactive interference at the memory level, enriching our understanding of the association between exercise and distractor suppression.

## 1 Introduction

Working memory (WM) is a mental workspace that enables the temporary maintenance and manipulation of information relevant to the task at hand (Baddeley, 2012; Carlisle et al., 2011). Due to its limited capacity, WM must prioritize task-relevant information while filtering out irrelevant content to support efficient interaction with complex visual environments (Cowan, 2010). This selective process is explained by the biased competition model of selective attention, which posits that information held in WM involuntarily biases visual selection toward matching stimuli in the visual field (Cowan, 2010). In line with this theory, many studies have demonstrated that memory-driven attentional capture can happen independently of the task relevance of the memory representation. In other words, attention can be obligatorily directed toward stimuli matching WM contents, even if those contents are irrelevant or even detrimental to the ongoing task (Jung et al., 2022; Kerzel and Schneider, 2025; Soto et al., 2008). For instance, Fu et al. (2021) adopted a WM/visual search dual-task paradigm where participants memorized the color of a shape and then searched for a tilted line among vertical distractors, each surrounded by colored shapes. The important manipulation was that these surrounding the distractor lines matched the memorized item in color, shape, both features, or neither. The results showed that, compared to the neutral condition, search reaction times (RT) were significantly longer under all matching conditions, along with an increase in overall fixation duration and a greater proportion of first saccades landing on distractors. These findings indicate that WM representations serve as an “attentional template,” exerting involuntary top-down control over attentional allocation (Gao et al., 2016).

However, there is evidence suggesting that attentional selection driven by WM representations is not entirely uncontrollable but is instead modulated by cognitive control (Kiyonaga and Egner, 2013; Whitehead et al., 2019). On the one hand, insufficient cognitive control increases susceptibility to memory-matching distractors, resulting in heightened attentional capture (Cai et al., 2024a). Several studies have demonstrated that individuals with impaired cognitive control struggle more to resist interference from memory-matching distractors, exhibiting more robust and earlier-onset attentional capture (Cai et al., 2022; Luo et al., 2021). For instance, Luo et al. (2021) found that the attentional capture effect was enhanced among individuals with high levels of anxiety, as anxiety negatively impacted top-down cognitive control mechanisms. On the other hand, adequate cognitive control can weaken memory-driven attentional capture or even reverse it into attentional suppression (Wen et al., 2018; Whitehead et al., 2019). Previous research has shown that WM representation can be strategically employed to divert attention away from memory-matching distractors when cognitive control is available with sufficient processing resources and time to engage in attentional selection processes (Gong et al., 2016; Han and Kim, 2009). For example, Han and Kim (2009) explicitly instructed participants that memory items would never serve as the search targets and delayed the visual search array. The results revealed that search RTs for invalid trials (where memory items matched search distractors) were significantly shorter than neutral trials (where memory items did not match any search items), demonstrating the presence of WM-guided distractor suppression. Furthermore, several studies have found that WM-guided suppression depends critically on sufficient processing time, allowing cognitive control to leverage WM representations, thereby actively suppressing memory-matching distractors (Cai et al., 2024b; Wen et al., 2018). These findings suggested that effective memory-driven attention relied on the availability of cognitive control.

Given that effective behavior in daily life emerges from the dynamic interplay between WM and attentional systems, it is crucial to explore how to enhance the efficiency of attentional capture driven by WM representations (Dowd et al., 2017). A recent study found that cognitive training intervention could effectively enhance top-down cognitive control to mitigate, though not eliminate, memory-driven capture (Sasin et al., 2022). In addition to cognitive training, an increasing amount of research has shown that physical exercise has a positive impact on cognitive function, with greater cognitive improvement for mind–body exercises that require cognitive tasks (Li et al., 2023; Uddin, 2021; Wang et al., 2024). Baduanjin (the Octupled Brocade), a traditional Chinese mind–body exercise, integrates physical movements, cognitive engagement, breathing regulation, and meditative techniques (Zheng et al., 2021). Many studies have found that baduanjin practitioners demonstrated superior performance on tasks requiring cognitive control—such as the Stroop task and Go/Nogo task—compared to sedentary controls, indicating that baduanjin has a promoting effect on cognitive control (Zhang et al., 2025; Zhou et al., 2025). For instance, Zhang et al. (2025) found that 16 weeks of baduanjin exercise led to enhanced cognitive control, manifested as improved NoGo accuracy, decreased N2 amplitude, and increased P2/P3 amplitudes in response to food-related NoGo stimuli. Neuroimaging studies have further shown that long-term baduanjin training is associated with enhanced functional connectivity in prefrontal-insular circuits, alongside structural increases in frontal and parietal gray matter volume, coupled with greater left prefrontal cortex recruitment during conflict processing (Chen et al., 2017; Tao et al., 2017; Zheng et al., 2021). These neurostructural and functional changes prove that baduanjin may enhance cognitive control by regulating the allocation of cognitive resources. In addition to long-term benefits, short-term mind–body exercises have been shown to improve inhibitory control and WM functions (Yao et al., 2022). Therefore, as an integrated mind–body practice, baduanjin appears to be a promising behavioral intervention for enhancing cognitive control through its multi-dimensional engagement of physical, cognitive, and meditative components (Xia et al., 2019).

Although the beneficial effects of baduanjin mind–body exercise on filtering perceptual interference by irrelevant distractors have been well documented, prior studies have not fully investigated whether such exercise also enhances resistance to distractor interference from WM. Addressing this research question is important, since individuals are frequently exposed to stimuli that inadvertently match contents actively held in WM, which can involuntarily capture attention and disrupt ongoing tasks (Soto et al., 2008). Elucidating how WM-driven attentional capture is modulated by long-term mind–body exercise would therefore help refine theoretical models of the dynamic interplay between WM and attentional control (Sasin et al., 2022), while offering a practical behavioral approach to optimize WM-driven attentional capture in distraction-rich environments.

Therefore, the present study aimed to determine the extent to which long-term baduanjin mind–body exercise modulates WM-driven attentional capture. A 16-week baduanjin intervention was implemented, and its training effects were assessed via a classic WM/visual search dual-task paradigm in which the match condition between memory items and search distractors was manipulated. Based on evidence that previous studies have found that short-term cognitive training intervention can enhance cognitive control to attenuate WM-driven capture (Sasin et al., 2022), together with findings that mind–body exercises strengthen cognitive control (Zhang et al., 2025), we hypothesized that 16 weeks of baduanjin practice would promote more efficient utilization of WM representations to direct attention away from memory-matching distractors, thereby mitigating or even eliminating distractor interference at the memory level. Specifically, we predicted a significant interaction effect of group (training vs. control) and test phase (pretest vs. posttest), such that the training group would exhibit a significant reduction in the magnitude of WM-driven attentional capture from pretest to posttest, whereas the capture effect in the control group would remain relatively stable.

## 2 Methods

### 2.1 Participants

A total of 67 volunteers were recruited via campus posters and online advertisements to participate in this study for monetary compensation or academic credit. The study used a pretest–posttest randomized controlled design with two parallel groups. To ensure a relatively balanced sample size between the two groups, participants were randomly assigned to either the training group (*n* = 34) or the control group (*n* = 33) using stratified randomization, with strata formed based on age, sex, and self-reported interest in mind–body exercise. Three participants from each group withdrew for personal reasons; thus, the final sample consisted of 61 participants (training group: *n* = 31, mean age = 19.59 ± 1.00 years, 11 men and 20 women; control group: *n* = 30, mean age = 19.52 ± 1.05 years, 7 men and 23 women). No significant differences were found in sex (*χ*^2^ = 1.08, *p* = 0.30), age (*t* < 1), and education (*t* < 1) between the training and control groups. We also conducted a power analysis prior to data collection using the G-power toolbox (Faul et al., 2009). The analysis indicated that a sample size of at least *N* = 16 per group would be sufficient to achieve the intended effect size (*f* = 0.25, representing a medium effect size, *α* = 0.05, power (1−*β*) = 0.80), ensuring adequate statistical power for the study. These estimated parameters aligned with findings from some previous exploratory studies in the field of memory-driven capture and exercise intervention (Cai et al., 2024b; Wang et al., 2023; Wen et al., 2018). All participants had no history of neurological or psychiatric disorders and had not participated in any regular mind–body exercise, such as Taichi, yoga, or qigong, in the past 3 years. Additionally, all participants reported normal (or corrected-to-normal) visual acuity and color vision and were right-handed, as confirmed by self-report and a brief behavioral verification (using the right hand for writing). All participants provided informed consent following the Declaration of Helsinki in advance of the study, and all experimental protocols were approved by the Ethics Committee of the School of Physical Education and Sports Science, Soochow University, China (Approval No. SUDA20250327H14).

### 2.2 Assessment and training protocol

The participants consisted of a training group and a control group. Each participant underwent a pretest consisting of a demographic questionnaire and a working memory guidance task within 5 days prior to training, and a posttest comprising a working memory assessment task within 3 days following training. The training group received 16 weeks of baduanjin (the Octupled Brocade) exercise training—a traditional Chinese mind–body exercise, while the control group did not receive the intervention. To ensure consistency in instruction and adherence, all participants in the training group attended the same group sessions in a dedicated studio, supervised by a skilled baduanjin instructor with 10 years of baduanjin experience. This intervention was administered at a frequency of 3 days/week and 60 min/session (Zhang et al., 2025). Each session was structured into a 5-min warm-up, 50-min of core training, and a 5-min cool-down. The core training of baduanjin involved a preparatory posture, eight core postures, and an ending posture, representing mind–body effectiveness (For details, see Chen et al., 2017; Tao et al., 2019). Both the participants and the instructors were blinded to the purpose of the study. To ensure participants’ exercise volume, the attendance rate of all participants was recorded. Participants were required to attend at least 85% of the total scheduled sessions to meet the adherence criterion. Regular verbal reminders and individual follow-ups were used to promote participation, and all sessions were held at a fixed time and location under the supervision of a certified instructor.

### 2.3 Working memory guidance assessment task

#### 2.3.1 Stimuli and apparatus

The experiment was designed and carried out using MATLAB 2018b (The MathWorks; Natick, MA) with the Psychophysics Toolbox (Brainard, 1997; Kleiner et al., 2007). Stimuli were delivered on a 16-inch LCD monitor (60 Hz, 1920 × 1,080 pixels) with a mid-gray background (RGB: 128, 128, 128), viewed from a distance of 57 cm in a dim and sound-attenuated laboratory room.

The experiment consisted of four displays: fixation display, memory display, search display, and probe display. The fixation display featured a black central fixation cross (1.03° × 1.03°). The memory and probe display each contained a centrally presented colored geometric shape (2.51° × 2.51°). The colors were randomly selected from a predefined set: green (RGB: 0, 128, 0), red (RGB: 255, 0, 0), yellow (RGB: 255, 255, 0), blue (RGB: 0, 0, 255), pink (RGB: 255, 192, 203). The shapes were also randomly chosen from the following five geometric shapes: circle, star, triangle, square, and hexagon. The search display was composed of four colored geometric squares (2.51° × 2.51°), arranged at equal distance on an imaginary central circle with a radius of 7.40°. These stimuli were placed at angles of either 30°/120°/210°/300° or 60°/150°/240°/330° relative to the horizon, with equal probability. Three of the search items contained a black vertical line (0.80° × 0.12°) as search distractors, while the remaining target item featured a black line tilted 12° either left or right from vertical. The color and shape in the search display were randomly selected from the set mentioned above, with each combination being unique.

#### 2.3.2 Design and procedure

**Figure 1:**
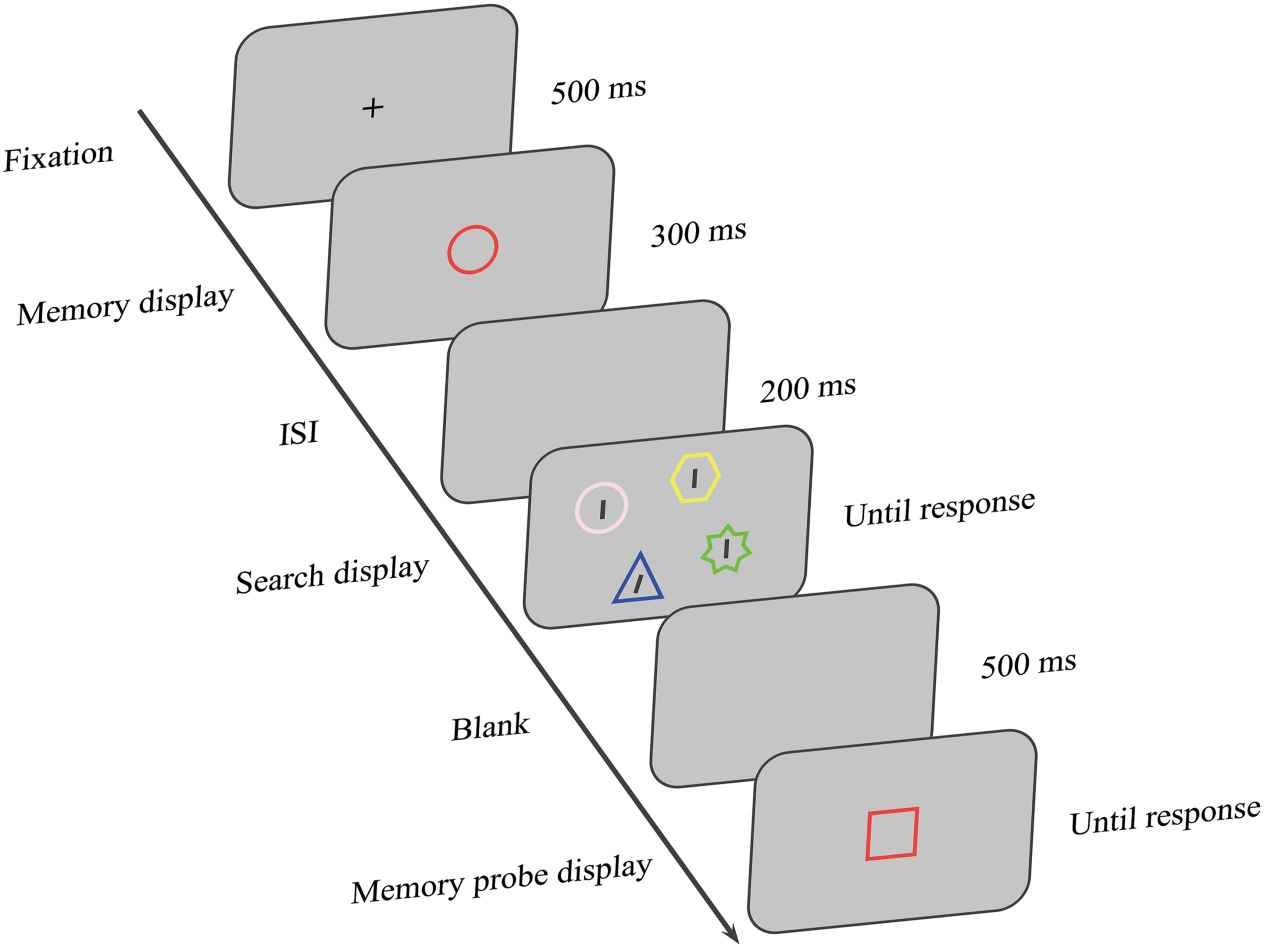
An example trial sequence. Participants were instructed to memorize the shape of a sample item, engage with a visual search task during the retention interval, and finally perform a shape change-detection test. The search display featured a distractor that could match the memory item in shape, color, both, or neither. Within this display, participants located a uniquely tilted line and reported its orientation.

The experiment integrated a WM task with an oblique feature search task. As illustrated in Figure 1, each trial started with a 500 ms central fixation cross “+,” during which participants were required to maintain fixation. The fixation cross was then replaced for 300 ms by a central colored shape. Participants were tasked with only memorizing its shape, preparing for the shape change detection test later. After a delay of 200 ms, a search display was presented, consisting of four colored geometric shapes with embedded black lines. To implement the four match conditions (shape, color, conjunction, neutral), the search display contained one distractor that shared the corresponding feature(s) with the memory item, while all other stimuli remained perceptually distinct. Participants were instructed to find the uniquely tilted target line and report its tilt direction (left/right) with a keypress, emphasizing both speed and accuracy. The display persisted until the response. After a 500-ms blank screen, memory was tested via a shape probe colored identically to the memory sample. Participants made a same/different judgment on its shape using the “F” or “J” key (50% same trials), focusing on accuracy. The inter-trial interval was 1,500 ms.

The formal experiment comprised a total of 140 trials, distributed across the different conditions: 56 trials in the neutral condition and 28 trials for each of the three match conditions. These trials were presented in four blocks of 35 trials each. Prior to the formal experiment, participants completed at least 16 practice trials identical in structure to the formal trials for familiarization. Participants needed to achieve an accurate rate exceeding 80% in both the search and memory tasks to proceed. To mitigate fatigue, self-paced rest breaks were provided between blocks, with subsequent blocks initiated by a keypress response from the participant. The entire session, including practice, lasted approximately 25 min.

#### 2.3.3 Data analysis

The accuracy rate (AR) and response time (RT) from the memory and search tasks were collated for analysis. For each participant’s search RT data, trials with errors or no responses in either task, as well as those with search RTs falling outside the range of condition-specific means ± 2.5 standard deviations, were excluded. The distribution of RTs was assessed by examining skewness and kurtosis. All skewness and kurtosis values fell within acceptable limits (absolute values < 3), indicating that the memory and search RT distributions could be regarded as sufficiently normal for parametric analyses (Kline, 2009, 2011).

Statistical analyses were performed using SPSS 27 (IBM Inc.) and followed a four-step analytical protocol: First, baseline characteristics (age, sex, education level) between the training and control groups were compared using independent samples *t*-tests and chi-square tests. Second, to assess the training effect on overall memory and search performance, a 2 (group: training, control) × 2 (test phase: pretest, posttest) × 4 (match condition: shape-match, color-match, conjunction-match condition, neutral condition) mixed factor ANOVA was conducted on mean search RTs, search ARs, memory RTs, and memory ARs, respectively. Greenhouse–Geisser correction was applied to the *p*-value for the ANOVA that violated sphericity, followed by multiple comparisons using the Bonferroni correction. Third, the memory-driven attentional guidance effects were calculated as the RT difference between the match condition (conjunction, shape, and color) and the neutral condition. Then, we conducted paired-sample *t*-tests to investigate how training modulates the strength of memory-driven attentional guidance in both groups. Fourth, independent-samples *t*-tests were conducted to assess group differences (training vs. control) in the magnitude of memory-driven attentional capture across the two testing phases (pretest and posttest).

## 3 Results

### 3.1 Memory and search ARs

The mean ARs across conditions for the memory and search tasks were summarized in Table 1. Both memory and search ARs reached ceiling levels (over 96 and 99%, respectively) under all conditions. A mixed-factor ANOVA was conducted on memory AR with test phase (pretest, posttest) and match condition (conjunction-match, shape-match, color-match, neutral) as within-subject factors and group (training, control) as a between-subject factor. The results revealed that the main effect of match condition was significant, *F*(2.41, 142.12) = 7.83, *p* = 0.048, η_p_^2^ = 0.05, with no other main effects or interactions reaching significance, *Fs* > 2.40, *ps* > 0.12. A mixed ANOVA on search AR showed that a significant effect of match condition, *F*(2.49, 146.78) = 2.93, *p* = 0.05, η_p_^2^ = 0.05, and no other significant effects, *Fs* > 2.20, *ps* > 0.11. These results indicated that participants reliably retained the memorandum and completed the visual search, unaffected by the group or the exercise intervention.

**Table 1:** Mean accuracy rates (AR, %) and standard errors as a function of group, test phase, and match condition for the visual search and/or working memory task.

Task	Test phase	Group	Match condition			
			Conjunction	Shape	Color	Neutral
Memory AR	Pretest	Training	98.04 ± 0.46	97.93 ± 0.57	97.81 ± 0.63	97.06 ± 0.53
		Control	97.50 ± 0.46	97.50 ± 0.67	96.90 ± 0.63	96.73 ± 0.48
	Posttest	Training	97.58 ± 0.65	97.81 ± 0.63	97.00 ± 0.62	96.89 ± 0.54
		Control	97.38 ± 0.68	96.67 ± 0.75	96.31 ± 0.79	96.13 ± 0.69
Search AR	Pretest	Training	99.88 ± 0.12	99.54 ± 0.22	99.19 ± 0.49	99.60 ± 0.30
		Control	99.40 ± 0.25	99.88 ± 0.12	99.64 ± 0.20	99.40 ± 0.23
	Posttest	Training	99.19 ± 0.27	99.88 ± 0.12	99.19 ± 0.32	99.71 ± 0.12
		Control	99.76 ± 0.17	99.88 ± 0.12	99.40 ± 0.25	99.35 ± 0.20

### 3.2 Memory RTs

The mean RTs for the WM task were calculated and submitted to a mixed ANOVA with test phase (pretest, posttest) and match condition (conjunction-match, shape-match, color-match, neutral) as within-subject factors and group (training, control) as a between-subject factor. The results revealed that the main effect of test phase was significant, *F*(1, 59) = 23.36, *p* < 0.001, η_p_^2^ = 0.28, memory RTs in the posttest (609 ms) were significantly shorter than those in the pretest (739 ms). The two-way interaction between test phase and group was also significant, *F*(1, 59) = 4.99, *p* = 0.029, η_p_^2^ = 0.08. For the group, simple effect analyses found that there was no significant difference in memory RTs between the two groups (747 vs. 730 ms) in the pretest, *t* < 1, but the training group (557 ms) showed significantly faster memory RTs than the control group (660 ms) in the posttest, *t*(59) = 3.11, *p* = 0.003, Cohen’s *d* = 0.80. Regarding the test phase, simple effect analyses found that the training group showed significantly faster memory RTs in the posttest (557 ms) compared to the pretest (747 ms), *t*(30) = 5.27, *p* < 0.001, Cohen’s *d* = 0.95. Similarly, the control group also demonstrated a marginal improvement in memory RT in the posttest (660 ms) compared to the pretest (730 ms), *t*(29) = 1.75, *p* = 0.073, Cohen’s *d* = 0.32. No other significant effects were found, *Fs* > 2.21, *ps* > 0.094. Based on these results, we concluded that long-term mind–body exercise enhanced the speed of memory retrieval.

### 3.3 Search RTs

The search RTs were analyzed in the same way as memory RTs. A mixed ANOVA with test phase and match condition as within-subject factors and group as a between-subject factor revealed a significant main effect of test phase, *F*(1, 59) = 26.31, *p* < 0.001, η_p_^2^ = 0.31, search RTs in the posttest (1,071 ms) were significantly shorter than those in the pretest (1,253 ms). The match condition was also significant, *F*(2.62, 153.39) = 14.97, *p* < 0.001, η_p_^2^ = 0.20, and *post hoc* comparisons with Bonferroni correction revealed that RTs were significantly shorter under the neutral condition (1,135 ms) than under the conjunction-match (1,189 ms), shape-match (1,159 ms), and color match conditions (1,165 ms), *ps* < 0.001. Moreover, the two-way interaction between test phase and group was also significant, *F*(1, 59) = 4.62, *p* = 0.036, η_p_^2^ = 0.07. For the group, simple effect analyses found that there was no significant difference in search RTs between the two groups (1,271 vs. 1,236 ms) in the pretest, *t* < 1, but the training group (1,012 ms) showed significantly faster search RTs than the control group (1,130 ms) in the posttest, *t*(59) = 2.52, *p* = 0.015, Cohen’s *d* = 1.16. Regarding the test phase, simple effect analyses found that the training group showed significantly faster search RTs in the posttest (1,012 ms) compared to the pretest (1,271 ms), *t*(30) = 5.93, *p* < 0.001, Cohen’s *d* = 1.07. Similarly, the control group also demonstrated a significant improvement in search RT in the posttest (1,130 ms) compared to the pretest (1,236 ms), *t*(29) = 1.88, *p* = 0.041, Cohen’s *d* = 0.34. No other significant effects were found, *Fs* > 1.19, *ps* > 0.31. This pattern of results indicated that long-term mind–body exercise improves the search speed.

**Figure 2:**
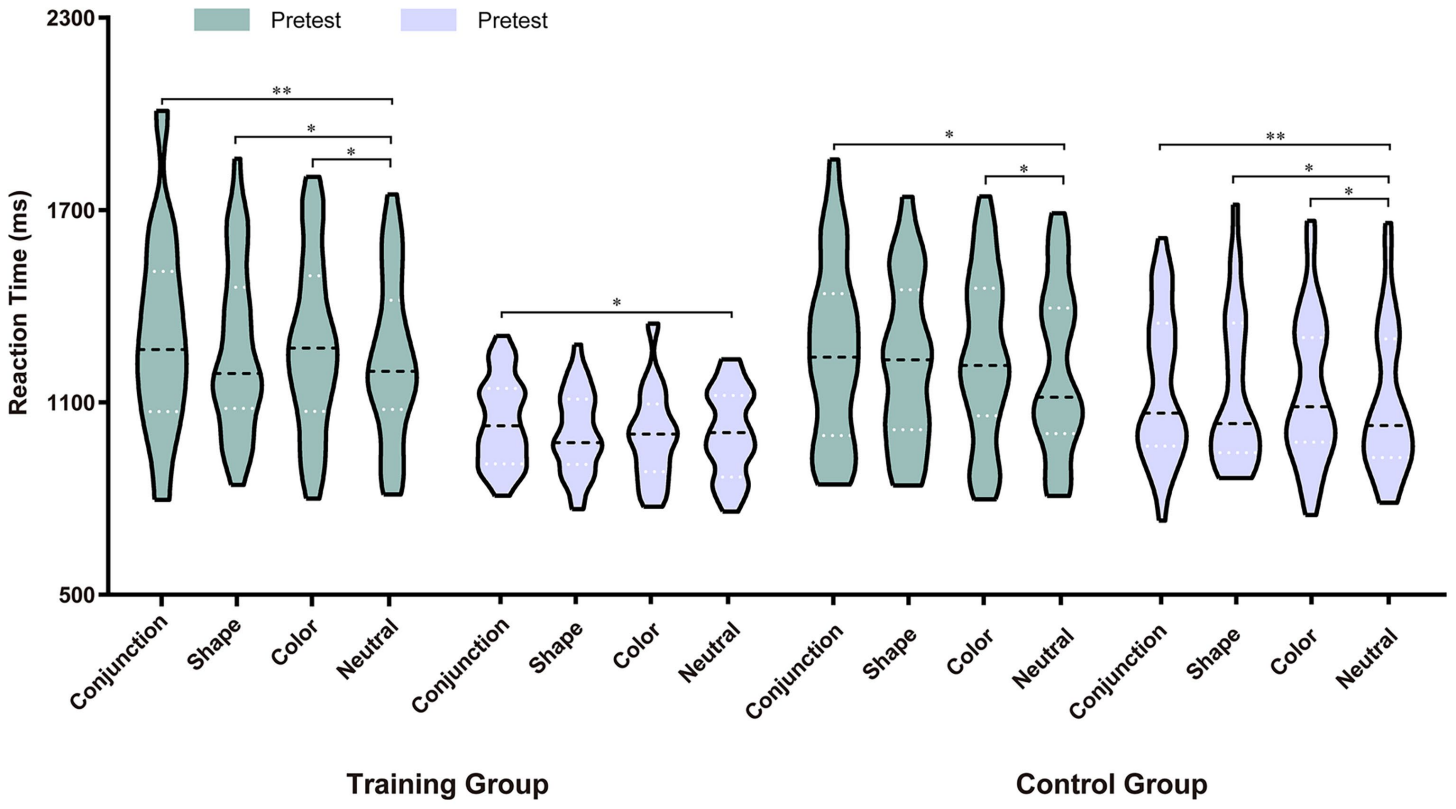
Search reaction times (RTs) are shown as a function of group, test phase, and match condition. The violin plot displays the data distribution, with the 75th percentile, median, and 25th percentile from top to bottom. *p < 0.05, **p < 0.01.

To determine whether memory-driven attentional capture existed in each group during each test phase, we conducted a one-way repeated measures ANOVA on the search RTs for both the training and control groups during the pretest and posttest phases, respectively. For the training group, during the pretest phase, there was a significant main effect of match condition, *F*(2.39, 71.83) = 6.48, *p* = 0.001, η_p_^2^ = 0.18. As shown in Figure 2, *post hoc* comparisons with Bonferroni correction revealed that RTs were significantly shorter under the neutral condition (1,232 ms) than under the conjunction-match (1,307 ms), *t*(30) = 3.86, *p* = 0.003, Cohen’s *d* = 0.69, shape-match (1,266 ms), *t*(30) = 2.93, *p* = 0.039, Cohen’s *d* = 0.53, and color match conditions (1,277 ms), *t*(30) = 2.87, *p* = 0.045, Cohen’s *d* = 0.52. During the posttest phase, there was a significant main effect of match condition, *F*(2.48, 74.32) = 3.67, *p* = 0.022, η_p_^2^ = 0.11. *Post hoc* comparisons with Bonferroni correction revealed that RTs were significantly shorter under the neutral condition (1,003 ms) than under the conjunction-match (1,038 ms), *t*(29) = 3.24, *p* = 0.018, Cohen’s *d* = 0.58, but not shape-match (1,005 ms), *t* < 1, and color match conditions (999 ms), *t* < 1. A paired-sample *t*-test was performed on the memory-driven attentional capture effect in the pretest and posttest under different feature conditions, respectively. The results showed that the attentional capture effects were smaller during the posttest phase than during the pretest phase for the conjunction (*t*(30) = 1.84, *p* = 0.075, Cohen’s *d* = 0.33), shape (*t*(30) = 1.98, *p* = 0.057, Cohen’s *d* = 0.36), and color (*t*(30) = 2.60, *p* = 0.014, Cohen’s *d* = 0.47).

On the other hand, for the control group, during the pretest phase, there was a significant main effect of match condition, *F*(2.19, 63.37) = 3.89, *p* = 0.022, η_p_^2^ = 0.12. As shown in Figure 2, post hoc comparisons with Bonferroni correction revealed that RTs were significantly shorter under the neutral condition (1,206 ms) than under the conjunction-match (1,262 ms), *t*(30) = 3.13, *p* = 0.024, Cohen’s *d* = 0.57, and color match conditions (1,246 ms), *t*(30) = 2.93, *p* = 0.040, Cohen’s *d* = 0.53, but not the shape-match condition (1,232 ms), *t*(30) = 1.98, *p* = 0.34. An independent sample *t*-test was performed on the memory-driven attentional capture effect for the training group and the control group under different feature conditions, respectively. The results showed no significant difference in attentional capture effects between the training group and the control group, regardless of the feature type, *ts* < 1, indicating that the baseline attentional capture effects were comparable between the two groups. During the posttest phase, there was a significant main effect of match condition, *F*(3, 87) = 4.62, *p* = 0.005, η_p_^2^ = 0.14. *Post hoc* comparisons with Bonferroni correction revealed that RTs were significantly shorter under the neutral condition (1,100 ms) than under the conjunction-match (1,149 ms), *t*(29) = 3.96, *p* = 0.003, Cohen’s *d* = 0.72, shape-match (1,135 ms), *t*(29) = 2.84, *p* = 0.049, Cohen’s *d* = 0.52, and color match conditions (1,139 ms), *t*(29) = 2.97, *p* = 0.036, Cohen’s *d* = 0.54. A paired-sample *t*-test was performed on the memory-driven attentional capture effect in the pretest and posttest under different feature conditions, respectively. The results showed that the attentional capture effects remained stable from the pretest phase to the posttest phase for these three types of features, *ts* < 1.

Additionally, we separately compared the memory-driven attentional capture effect for the training group and the control group during the pretest and posttest phase under different feature conditions. The results showed that the attentional capture effects were significantly smaller among the training group than the control group for both the shape (*t*(59) = 2.08, *p* = 0.042, Cohen’s *d* = 0.53) and color (*t*(59) = 2.32, *p* = 0.024, Cohen’s *d* = 0.60) during the posttest phase. However, no significant between-group differences in attentional capture effects were observed for conjunction during the posttest phase, *t* < 1, and the three types of features during the pretest phase, *ts* < 1. These results indicated that the memory-driven attentional capture effect decreased or even disappeared among the training group after 16 weeks of exercise intervention, while the same effect remained unchanged among the control group, which did not undergo the intervention.

## 4 Discussion

Employing a WM/visual search dual-task paradigm, the present study investigated how 16 weeks of baduanjin exercise intervention influences memory-driven attention. The training group participated in pre- and post-intervention assessments and engaged in regular baduanjin practice throughout the intervention period, while the control group followed the same testing schedule without undergoing the exercise training. The results revealed that the training group demonstrated significantly faster search and memory RTs during the posttest phase relative to both their own pretest performance and the posttest performance of the control group. More importantly, relative to the control group, which maintained stable attentional capture effects from pretest to posttest, the training group demonstrated a significant reduction in WM-guided attentional capture, encompassing both object-based and feature-based capture. Furthermore, feature-based attentional capture was significantly smaller among the training group than among the control group during the posttest phase. These findings suggest that 16 weeks of baduanjin exercise can improve memory-driven attentional capture and enhance the efficiency of both visual search and memory retrieval processes.

It was found that during the pretest phase, both groups exhibited slower search RTs under all matching conditions compared to the neutral condition, indicating the presence of a WM-driven attentional capture effect. These results align with object-based encoding theory, which posits that both task-relevant and task-irrelevant features of an object are integrated into a unified feature template and collectively influence attentional selection at the object level (Foerster and Schneider, 2020). Recent research further suggests that encoding a relevant feature promotes the prioritization of all features belonging to that object for visual processing and attentional selection, a process driven by an involuntary, object-based mechanism (Kerzel and Schneider, 2025; Shi et al., 2025). For instance, Jung et al. (2022) demonstrated that stimuli matching either task-relevant or -irrelevant features captured attention across different visual search tasks (e.g., binary-stimulus search or unitary stimulus search). Our results critically extend previous research by demonstrating that both object-based and feature-based attentional capture effects remain stable over a 16-week period, as evidenced by the control group’s consistent search performance from the pre- and post-tests. However, one issue remains to be addressed: although the control group showed slower search RTs under the shape-match condition compared to the neutral condition during the pretest phase, this difference was not statistically significant, suggesting a lack of reliable shape-based attentional capture. This may be due to the relatively brief memory display duration (500 ms) employed in this study, combined with the inherently weaker attentional capture capacity of shape features, resulting in unstable capture effects (Gao et al., 2016; Li et al., 2024). Indeed, studies using similar temporal parameters have also failed to observe significant attentional capture by shape (Li et al., 2024).

Importantly, the current results showed that the memory-driven attentional capture effect was significantly reduced among the training group during the posttest phase compared to both their own pretest performance, and was significantly smaller than that observed among the control group during the posttest phase. Specifically, following 16 weeks of baduanjin exercise, the training group exhibited decreased object-based attentional capture, while capture effects based on both relevant shapes and irrelevant colors were no longer observed. Building upon previous research findings that long-term physical and mental exercise enhances WM and selective attention (Luo et al., 2024; Tao et al., 2017; Ye et al., 2024), the current results further demonstrate that these benefits extend to memory-driven capture processes. One might argue that the reduction of memory-driven attentional capture found among the training group was caused by the overall faster search RTs during the posttest phase. That is, training accelerated search speed, thereby enhancing the ability to resist interference from memory-matching distractors. However, previous studies suggest that search speed differentially modulates memory-driven attentional capture (Cai et al., 2024b). Han and Kim (2009) divided participants into fast and slow groups based on neutral-trial RTs, and found that the fast group exhibited significant attentional capture, whereas the slow group showed the opposite attentional suppression—a pattern attributed to slower responses may allow cognitive control mechanisms to engage and inhibit distracting information. Therefore, the attenuation of memory-driven attentional capture we observed among the training group is unlikely to be attributable to fast search speeds alone.

Prior research has manipulated a range of cognitive control-related variables, such as reward association (Gong et al., 2016), delayed search array presentation (Han and Kim, 2009), directed forgetting (Sasin et al., 2017), and multi-day reinforcement training (Sasin et al., 2022), all of which have been shown to effectively attenuate attentional capture and, in some cases, even reverse it into attentional suppression (see also Won and Zhang, 2025). Therefore, one plausible mechanism underlying the beneficial effect of baduanjin exercise on the guidance of attention by WM is that long-term exercise enhances cognitive control, thereby actively suppressing attention to memory-matching distractors and facilitating visual search efficiency. Supporting this view, studies have reported that baduanjin practitioners exhibit superior cognitive performance on conflict tasks such as the Stroop, Go/No-go, and Flanker tasks, further confirming its role in strengthening cognitive control (Chen et al., 2017; Yao et al., 2022; Zhou et al., 2025). Baduanjin may enhance cognitive control through two pathways. On the one hand, as a low-to-moderate intensity aerobic activity, baduanjin exercise integrates substantial cognitive engagement—such as task switching, updating, and inhibition of irrelevant actions and thoughts—during practice (Gong et al., 2025; Wang et al., 2021). Aerobic exercise, particularly that which requires high cognitive demand, has been recognized as an effective means to improve cognitive control (Kao et al., 2022; Yang et al., 2025). Furthermore, prior research has demonstrated that 4 weeks of cognitive training can effectively mitigate but not eliminate memory-driven attentional capture by enhancing cognitive control (Sasin et al., 2022). Therefore, considering that the training group exhibited no feature-based attentional capture after the intervention, the current study appeared to indicate that dual training, which combines physical and cognitive elements, leads to greater enhancements in resisting proactive interference at the memory level compared to training that focuses solely on cognitive elements. On the other hand, as a mind–body exercise, baduanjin combines physical, cognitive, and meditative elements within a single activity, emphasizing the cultivation of both proprioceptive and interoceptive attention (Xia et al., 2019). Systematic reviews and meta-analyses have concluded that mindfulness meditative training enhances executive control processes—including inhibition, working memory, and cognitive flexibility—by both expanding available cognitive resources and optimizing their allocation (Gallant, 2016; Cásedas et al., 2020). Neuroimaging evidence further indicates that intensive mindfulness meditation modulates activation in cognitive control regions and strengthens functional connectivity between the frontoparietal regions and the dorsolateral prefrontal cortex (Taren et al., 2017; Yordanova et al., 2021). Thus, baduanjin mind–body exercise may enhance cognitive control over WM-driven attentional capture, potentially through the expansion of the attentional resource pool and more strategic resource allocation. These potential mechanisms warrant further empirical investigation.

Furthermore, the current data showed that the training group had significantly shorter memory RTs during the posttest phase compared to the pretest phase. This improvement could not be attributed to practice effects, as memory RTs among the training group were also significantly shorter than those among the control group during the posttest phase. These findings support the growing body of evidence suggesting that long-term exercise can facilitate memory retrieval (Ye et al., 2024). Recent work has demonstrated that different forms of exercise exert distinct effects on WM, with aerobic exercise primarily optimizing memory retrieval speed (Guo et al., 2025). According to the memory strengthening theory, both memory accuracy and time are key indicators of the strength of information storage representation, and when accuracy approaches ceiling levels, memory RT becomes an especially crucial measure (Kahana and Loftus, 1999; Yu et al., 2022). Thus, the observed reduction in memory RTs following baduanjin exercise may reflect enhanced strength or accessibility of memory representations. This interpretation is consistent with the view proposed by Kiyonaga and Egner (2013) that memory and attention processes draw upon a shared pool of cognitive resources; therefore, more efficient memory encoding may free up resources available for subsequent cognitive operations, such as memory-driven capture and memory retrieval. Several studies have found that cognitive control over memory-driven capture is implemented, at least in part, through altering the state of memory representations (Cai et al., 2024b; Gong et al., 2016; Kiyonaga et al., 2012). Taken together, these findings suggest that 16 weeks of baduanjin exercise may enhance WM representations and synergize cognitive control to optimize both search and memory performance.

## 5 Limitations and implications

There are several limitations to the present study. First, the beneficial effects of baduanjin exercise are attributed to enhanced cognitive control, which is inferred from behavioral performance patterns in a well-validated WM/visual search dual-task paradigm, in which resistance to memory-driven attentional capture is considered a behavioral index of top-down cognitive control (Han and Kim, 2009; Sasin et al., 2022; Whitehead et al., 2019). Future studies incorporating classic cognitive control tasks and neurophysiological techniques (such as electroencephalography or transcranial electrical stimulation) would help to further specify the underlying mechanisms. Second, the control group in this study did not receive an active intervention. While this design allows us to evaluate the effects of baduanjin mind–body exercise against a natural baseline, it cannot rule out the potential influence of non-specific factors such as participant expectancy, regular social contact with the instructor and peers, or the simple physical activity (Chen et al., 2021; Zhang et al., 2025). In the future, randomized and active controlled conditions (e.g., a light physical exercise or stretching program) should be designed to examine whether the observed benefits are unique to the mind–body components of baduanjin exercise. Finally, our sample was predominantly composed of young university students, resulting in a narrow age range and a relatively homogeneous educational background. Consequently, the generalizability of the current findings to other age groups, individuals with different educational experiences, or clinical populations may be limited. Future research should examine whether the influence of baduanjin mind–body exercise on memory-driven attention extends to more diverse populations.

Despite these limitations, the current findings advance our understanding of WM-driven attentional capture by showing that long-term mind–body exercise contributes to attentional capture from both memory-matching and non-matching distractors. The results support the view that although the maintenance of information in WM creates a strong attentional bias (Desimone and Duncan, 1995), the interaction between WM and attention is dynamic and can be modulated by various factors that engage cognitive control, such as mind–body exercise (Gong et al., 2016; Han, 2015; Sasin et al., 2022). This sets a new foundation for future research aimed at uncovering the intricate mechanisms that contribute to these positive effects. Additionally, this may have important practical implications. For instance, incorporating moderate mind–body exercises during class breaks within schools could help students resist memory-based proactive interference, potentially supporting classroom focus and academic performance. Furthermore, given that individuals with cognitive control deficit show heightened susceptibility to proactive interference (Cai et al., 2022; Cai et al., 2024a; Loosli et al., 2014), mind–body practice training may offer a feasible behavioral intervention to strengthen their ability to filter more proactive interference from WM.

## 6 Conclusion

While prior research was mainly concerned with the beneficial effects of physical exercise in filtering external perceptual distractors, the present study focused on whether these benefits could extend to another critical aspect of inhibition function—resistance to proactive interference from the internal WM. By implementing 16 weeks baduanjin mind–body exercise, in conjunction with classic WM/visual search dual-task paradigm, the present study demonstrates that mind–body exercise not only promoted search and memory retrieval speeds, but more importantly, reduced attentional capture driven by memory-matching distractors, possibly due to enhanced cognitive control. These findings advance theoretical views regarding the flexibility of the interplay between WM and attentional control, and suggest that baduanjin could serve as a practical intervention to strengthen WM distraction resistance in everyday contexts.

## Data Availability

The raw data supporting the conclusions of this article will be made available by the authors, without undue reservation.
